# Implante de 5,5 mm. Una solución para atrofias severas sin renunciar a la predictibilidad

**DOI:** 10.21142/2523-2754-1004-2022-137

**Published:** 2023-12-26

**Authors:** Eduardo Anitua

**Affiliations:** 1 Private practice in oral implantology, Eduardo Anitua Institute, Vitoria, Spain. eduardo@fundacioneduardoanitua.org Private practice in oral implantology Eduardo Anitua Institute Vitoria Spain eduardo@fundacioneduardoanitua.org; 2 Clinical researcher, Eduardo Anitua Foundation, Vitoria, Spain. Clinical researcher, Eduardo Anitua Foundation Vitoria Spain; 3 University Institute for Regenerative Medicine and Oral Implantology - UIRMI (UPV/EHU-Fundación Eduardo Anitua), Vitoria, Spain. University Institute for Regenerative Medicine and Oral Implantology - UIRMI (UPV/EHU-Fundación Eduardo Anitua) Vitoria Spain

**Keywords:** implante corto, atrofia ósea, elementos finitos, short dental implant, bone atrophy, finite element model

## Abstract

La rehabilitación de zonas de maxilar y mandíbula con extrema reabsorción es un hecho de mayor presencia en nuestra consulta dental. Contar con técnicas quirúrgicas como los implantes cortos y extracortos nos facilitan la resolución de estos casos clínicos. En el presente reporte de caso clínico, desarrollamos un caso rehabilitado mediante un implante de 5,5 mm de longitud y aportamos un estudio biomecánico del comportamiento de implantes de estas características.

## Abstract (en)

The rehabilitation of areas of the maxilla and mandible with extreme resorption is a fact of increased presence in our dental practice. Surgical techniques such as short and extra-short implants facilitate the resolution of these clinical cases. In this clinical case report we develop a case rehabilitated using a 5.5 mm long implant and we provide a biomechanical study of the behaviour of implants with these characteristics.

## INTRODUCCIÓN

Los pacientes con atrofia ósea marcada tanto en horizontal como en vertical son cada vez más numerosos en la consulta dental. Por ello, los implantes cortos y extracortos son cada vez más utilizados en los tratamientos de rehabilitación. Los implantes cortos son ya una más de las técnicas de rehabilitación del maxilar atrófico que podemos considerar “de rutina”, por ser una opción mínimamente invasiva y con cifras de supervivencia en torno al 99%^1^^,^^2^. 

Aun así, existen casos en los que estos implantes no pueden insertarse de forma directa sin el empleo previo de técnicas accesorias de regeneración ósea que nos permitan recuperar el volumen óseo perdido ^3^^-^^6^. Para solventar estas situaciones de forma mínimamente invasiva, nacen los implantes 4,5 mm de longitud. Los implantes cortos dieron paso a los extracortos y hoy en día disponemos de implantes que podemos denominar ultracortos, diseñados con longitudes de 4,5 y 5,5 mm, los cuales son capaces de ser rehabilitados sin incremento de las tasas de fracaso, comparados con otros implantes de longitud mayor ^11^^-^^15^. Con esta nueva longitud podemos afrontar nuevos retos en mandíbula y maxilar con altura ósea residual menor de 5 mm sin necesidad de aplicar técnicas accesorias ^16^^,^^17^. 

Estos implantes, por su parte, sirven como excelente solución en las atrofias verticales, al evitar técnicas regenerativas accesorias, así como cirugías con mayor morbilidad para los pacientes ^12^^-^^14^. A pesar de ser un implante con menor experiencia clínica, los resultados mostrados en la literatura internacional para los implantes de 4 mm son comparables a los obtenidos mediante técnicas regenerativas para aumentar el volumen óseo y la inserción posterior de implantes de longitud “convencional” ^8^^-^^11^. Una de las principales dudas que podemos tener respecto del uso de estos implantes es el comportamiento biomecánico y de generación de tensiones en el hueso crestal, debido a su escasa longitud, además de su capacidad para generar una oseointegración similar a la de los implantes con mayor longitud y superficie de integración. 

El comportamiento biomecánico de estos implantes ha sido analizado por nuestro grupo de estudio en función de la longitud y el diámetro, y no se observan grandes diferencias en lo que a la longitud se refiere, una vez que el implante se encuentra perfectamente integrado, al trabajar únicamente las primeras espiras. Podemos pensar, entonces, que la superficie reducida de titanio de estos implantes podría ser un impedimento para una correcta oseointegración si los comparamos con implantes con mayor superficie en contacto con el hueso receptor. 

Este hecho no debería tener importancia si la superficie que se utiliza en el implante facilita la integración y la estabilidad primaria inicial. La superficie de los implantes BTI de 4,5 mm de longitud (UniCca®), resulta de la incorporación a la superficie multirrugosa óptima de una capa de iones de calcio. Esta modificación química, higroscópica y polar convierte la superficie en superhidrofílica, lo que implica el contacto completo de la sangre y el plasma con todos los puntos de la superficie, incrementando al máximo la superficie activa para la regeneración. Ya desde el posicionamiento del implante en el lugar de implantación, la superficie se recubre automáticamente por capilaridad ^18^^,^^19^.

Los estudios celulares con osteoblastos primarios obtenidos con consentimiento a partir de hueso de pacientes sometidos a cirugía oral revelan que la superficie modificada con calcio UnicCa permite una mayor adhesión y proliferación de los osteoblastos, así como los induce a una mayor síntesis de matriz extracelular ^18^. En los estudios realizados en animales, esta superficie de implantes ha mostrado una diferencia significativa en el contacto implante-hueso comparado con la superficie sin tratamiento de calcio (óptima) ^18^. Al realizarse una modificación de la superficie que facilita su integración, se reduce el riesgo de que la disminución de la superficie activa pueda generar algún tipo de repercusión negativa al ser insertado el implante. Como en implantes de otras longitudes, el hecho de ser conservador con el lecho donde se insertan es crucial; por ello, la realización de un protocolo cuidadoso de fresado (fresado biológico) y la adaptación del neoalveolo al implante evita puntos de compresión que generen isquemia y posible reabsorción ^20^^-^^21^.

En el siguiente trabajo mostramos la biomecánica de estos implantes ante diferentes supuestos de carga mediante la técnica de elementos finitos, además de ilustrar su eficacia clínica mediante la exposición de un caso clínico rehabilitado con un implante de 5,5 mm. 

## PRESENTACIÓN DEL CASO

El presente reporte de caso utiliza los elementos finitos para convertir una estructura en un número finito de partes llamadas elementos, cuyo comportamiento se especifica con un número finito de parámetros. Dichos elementos contienen una serie de números interconectados entre sí llamados nodos y al conjunto se le conoce como malla. Este método de análisis permite estimar con gran precisión y simplicidad los esfuerzos y deformaciones que va a sufrir una pieza o un conjunto de piezas al ser sometidas a un sistema de cargas ^22^^,^^23^. 

Esta técnica permite, por lo tanto, realizar la evaluación de los diseños de diferentes implantes simulando la dispersión de la tensión oclusal en distintos supuestos, con un implante integrado en el lecho óseo. Con ellos se reproducen las distintas variables que intervienen en el conjunto: hueso, implante, pilar intermedio (si está presente) y dispersión de la tensión en el hueso con distinta densidad, y se puede ajustar los diferentes parámetros para obtener resultados en función de los supuestos que se analizan. En nuestro caso, hemos analizado la dispersión de tensiones hacia el hueso crestal de un implante de 4,5 mm de longitud y 3,5 mm de diámetro (plataforma interna estrecha -Core®), comparado con implantes de mayor longitud y mismo diámetro (5,5 y 11 mm), para comprobar si se generan mayores picos de tensión con la longitud de 4,5 mm y, de ese modo, conocer si existe un mayor riesgo de pérdida ósea o fracaso de la integración del implante. El tipo óseo es el considerado estándar (tipo III-IV) y en todos los supuestos se emplea una pieza intermedia (transepitelial unitario de 2 mm de longitud). La tensión aplicada al conjunto es una carga vertical a 200 Ncm para los tres supuestos. Las tensiones máximas obtenidas en cada supuesto se compararon dos a dos mediante t de Student y las tres entre sí mediante análisis de la varianza, estimándose que existían diferencias estadísticamente significativas si p<0,05 (SPSS v15.0, SPSS Inc. Chicago, Il, EE. UU.). 

## RESULTADOS

**Figura 1:**
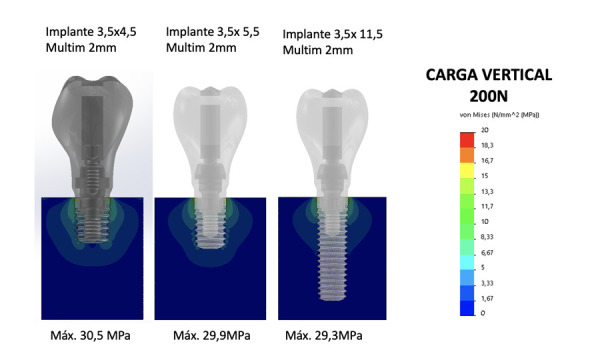
Imágenes de distribución de tensiones con carga vertical a 200 Ncm en los tres supuestos estudiados.

La diferencia de tensión máxima recibida por el hueso entre el implante de 5,5 mm de longitud y el de 4,5 mm de longitud fue de 0,6 megapascales (Mpa), sin diferencias estadísticamente significativas entre ambos casos (p = 0,227). La diferencia de tensión máxima entre el implante de 4,5 mm y el de 11,5 mm fue de 1,2 Mpa, igualmente sin diferencias significativas (p = 0,309). Al comparar la tensión máxima recibida por el lecho óseo entre los tres supuestos entre sí, tampoco se objetivaron diferencias estadísticamente significativas (p = 0,229). La distribución de las tensiones recibidas en todos los supuestos se concentra en las dos primeras espiras, siendo por debajo de las mismas cercana a 0, como puede verse en la Figura 1. En las imágenes 2 a 12 mostramos un caso clínico rehabilitado mediante el implante de 4,5 mm y su comportamiento clínico al ser sometido a carga, tal como se ha simulado en el modelo de elementos finitos. 

**Figuras 2 y 3:**
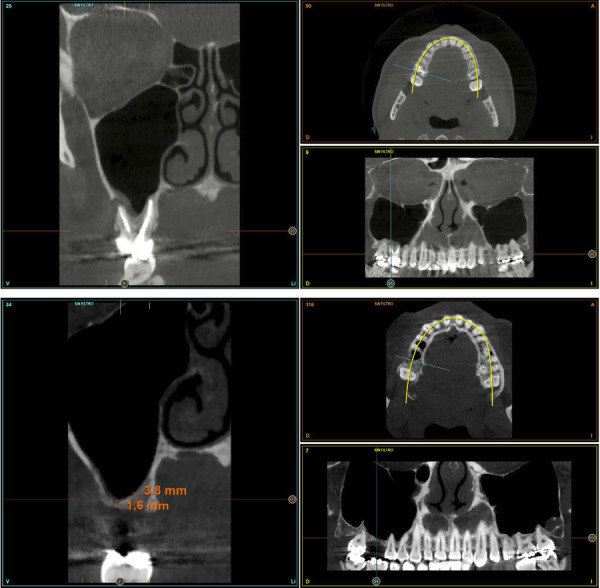
Imagen inicial del *cone-beam* con el molar situado en posición 16 con un quiste apical que precisa ser extraído. En el corte de control tras la exodoncia y regeneración del alveolo con Endoret-PRGF nos muestra una altura de 3,5 mm en la zona de mayor volumen (área palatina).

**Figura 4:**
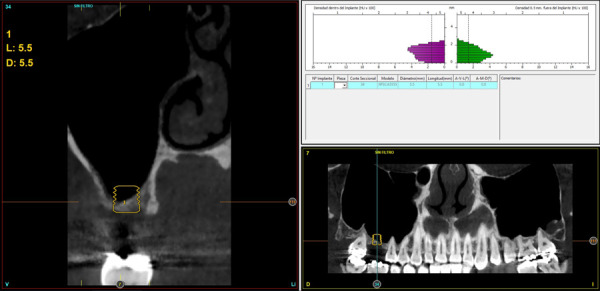
Imagen de planificación del *cone-beam* con el implante de 5,5 mm de altura y 5,5 mm de anchura. Podemos observar como con esta anchura podemos lograr un correcto anclaje vestíbulo-palatino. En el área vestibular, se realizará una pequeña elevación de seno transalveolar para ganar el volumen óseo necesario.

**Figuras 5 y 6:**
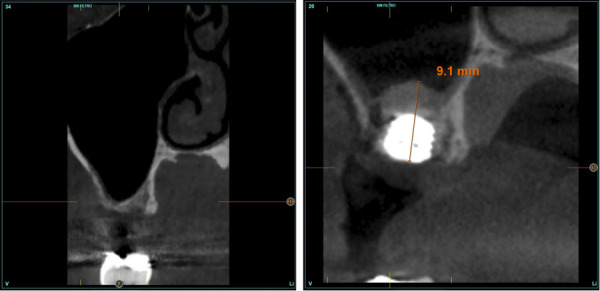
Imagen inicial (antes de la colocación del implante) y final tras la inserción y elevación de seno.

**Figuras 7 y 8:**
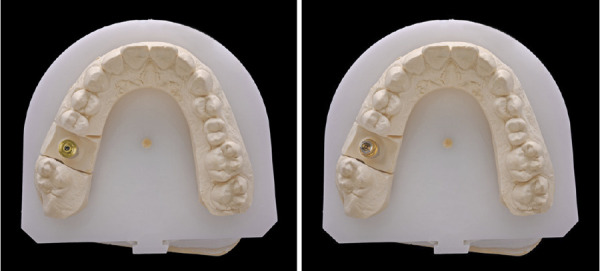
Imágenes de la confección de la corona con transepitelial unitario y el uso de la interfase sobre el mismo.

**Figuras 9 y 10:**
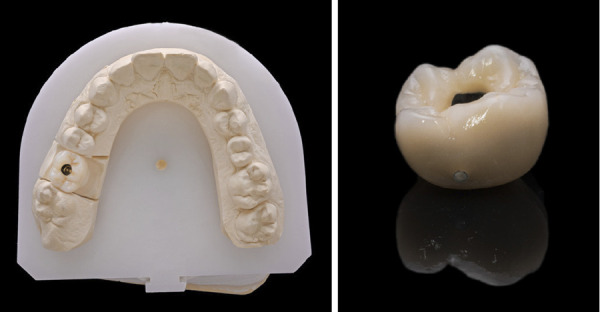
Corona definitiva lista para ser cementada en la interfase.

**Figuras 11 y 12:**
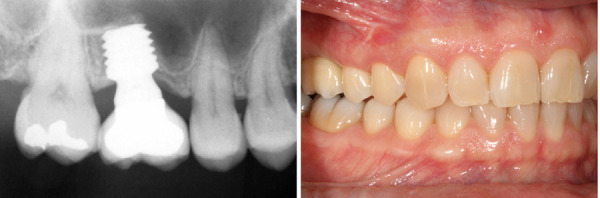
Imagen radiológica al año de seguimiento, donde observamos la estabilidad del implante y del tejido blando. Podemos observar también como existe una mayor densificación del hueso alrededor del implante tras la carga y función.

## DISCUSIÓN

El uso de los implantes de menor longitud para casos de atrofias extremas en sentido vertical es una técnica mínimamente invasiva, con muy buenos resultados y cada vez más extendida para el abordaje de este tipo de casos ^1^^,^^25^^-^^27^. 

El principal problema que enfrentamos con los implantes ultracortos, como el presentado en este caso, de 5,5 mm de longitud, es la necesidad de lograr una estabilidad primaria en un área muy reducida de hueso remanente, por lo que el estudio en profundidad del lecho óseo y la orientación de la secuencia de fresado a este estudio es primordial, así como el uso de una superficie que nos facilite la integración inmediata ^19^^-^^21^. 

En los estudios realizados en animales, esta superficie de implantes ha mostrado una diferencia significativa en el contacto implante-hueso comparado con la superficie sin tratamiento de calcio (óptima) ^19^. En este estudio, los implantes insertados mediante esta técnica con la superficie UnicCa han mostrado una correcta oseointegración, a pesar del volumen óseo remanente (al límite para la realización de una elevación transalveolar con éxito) y han favorecido la neoformación ósea intrasinusal sin el empleo de biomateriales para el relleno del seno, usándose únicamente Endoret-PRGF. La estabilidad del implante es crucial para su integración, por lo que, con implantes ultracortos, como el presentado en este caso, existe la necesidad de lograr una estabilidad primaria en un área muy reducida de hueso remanente. Esta estabilidad inicial puede ser lograda, como hemos reportado en este artículo, a pesar de que existan condiciones desfavorables (baja altura ósea y baja densidad) con implantes que bicorticalizan vestíbulo-lingualmente, como el caso del implante de 5,5 mm de longitud empleado. Una vez el implante ha sido insertado y se ha integrado, la distribución de tensiones generará una densificación ósea alrededor del implante que mantendrá la estabilidad del tratamiento. 

Podemos considerar, por lo tanto, a este grupo de implantes de 4 y 4,5 mm como una alternativa más a los procedimientos de regeneración ósea para mandíbulas y maxilares con extrema atrofia vertical, pues se logra con ellos un abordaje mínimamente invasivo de los casos y se obtenien resultados similares o, en algunos casos, mejores que con las técnicas convencionales de recuperación del volumen óseo residual y la inserción de implantes de mayor longitud ^7^^,^^10^. 

## CONCLUSIONES

El uso de implantes de 5,5 mm puede ser una estrategia eficaz para las atrofias óseas verticales. Como en la mayoría de los casos complejos, cuando nos enfrentamos a estas situaciones, la planificación cuidadosa y la secuencia de fresado, así como el empleo de superficies que favorezcan la integración pueden marcar la diferencia. Debemos tener en cuenta que, una vez el implante se encuentre integrado, una oclusión correcta realizada con una prótesis que garantice el ajuste pasivo y la estanqueidad, como la aportada en este trabajo con transepitelial unitario, puede ayudar a mantener el resultado logrado. No debe preocuparnos el rendimiento biomecánico a largo plazo del implante de 5,5 mm al no existir diferencias significativas en la transmisión de cargas al hueso, tal como hemos mostrado con el estudio de elementos finitos.

## Fuente de financiamiento:

Autofinanciado
